# Supplementary material for: The Distinctive Permutated Domain Structure of Periplasmic α-Amylase (MalS) from Glycoside Hydrolase Family 13 Subfamily 19
Source: Molecules. 2023 May 9;28(10):3972. doi: 10.3390/molecules28103972 (PMC10223318; doi:10.3390/molecules28103972)
Supplement: Supplementary file 1 [file molecules-28-03972-s001.zip › molecules-2345953-supplementary.pdf]

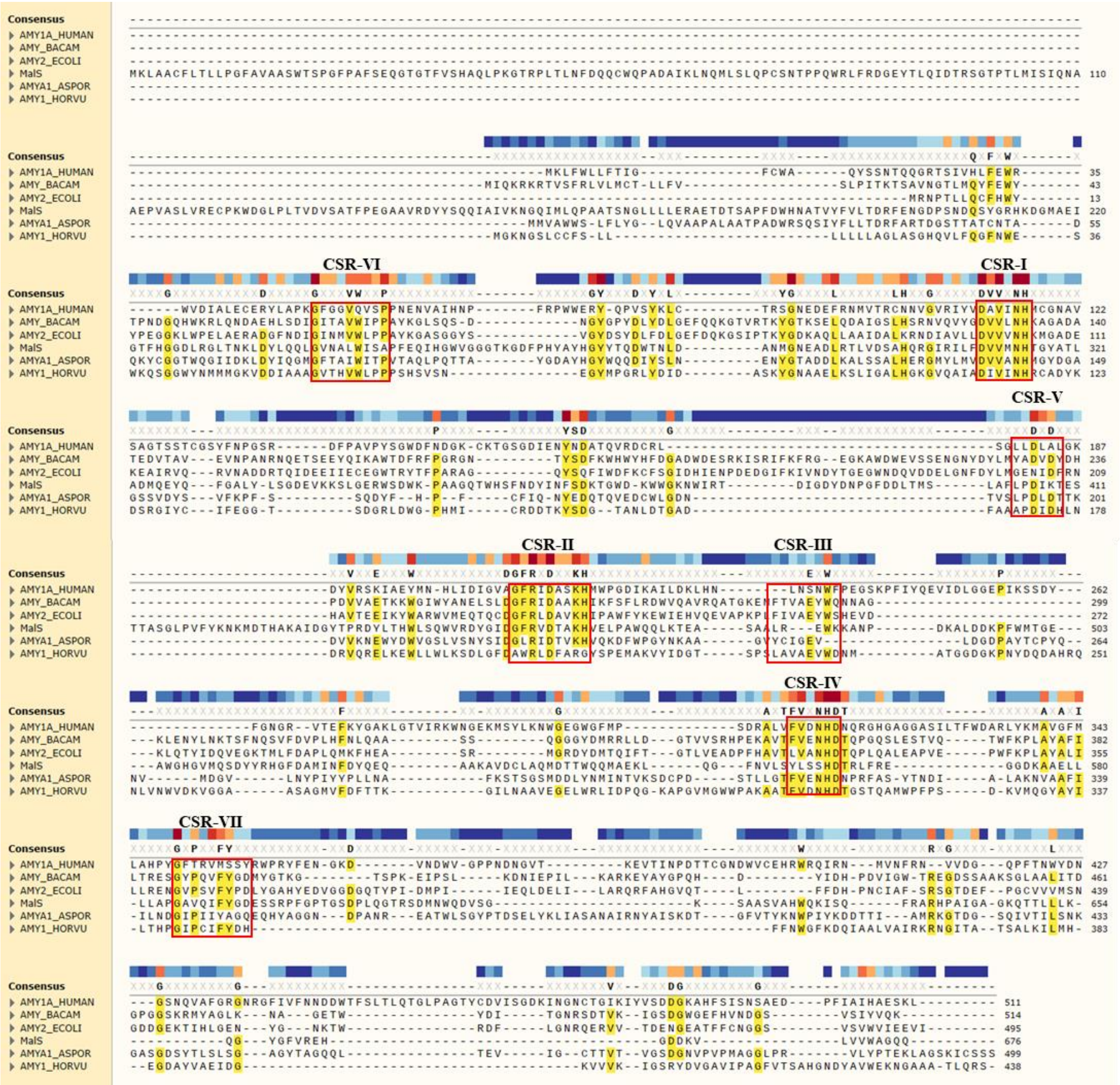

Figure S1: Multiple sequence alignment of α-amylases. Multiple sequence alignment of MalS, AMY1A_HUMAN (Homo sapiens, human), AMYA1_ASPOR (from *Aspergillus oryzae* strain ATCC 42149/ RIB 40), AMY_BACAM (*Bacillus amyloliquefaciens*), and AMY2_ECOL1 (Cytoplasmic alpha- amylase, *Escherichia coli* K12), AMY_HORVU (Hordeum vulgare). The conserved sequence regions (CSRs) are shown in the red squares.

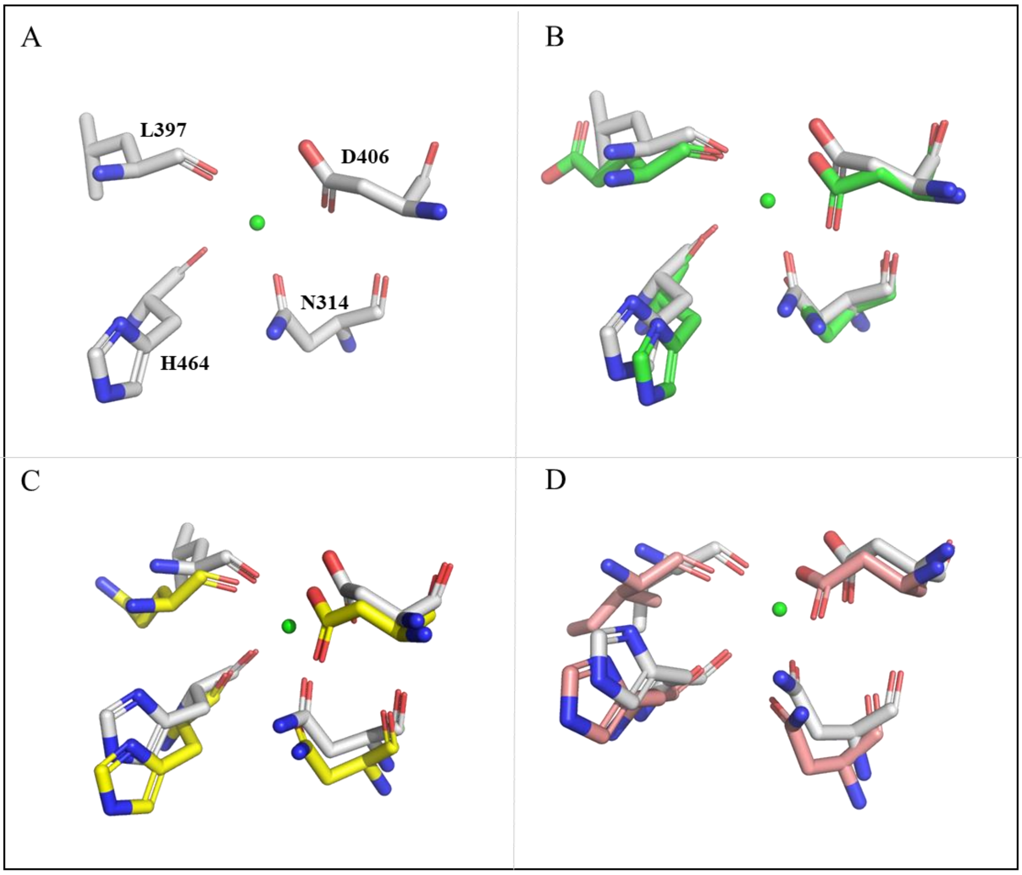

Figure S2A: Ca1 residues alignment. (A) Ca1 interacts with residue of MalS; (B) MalS and *Anoxybacillus ayderensis* α-amylase(5a2a) alignment; (C) MalS and *Geobacillus stearothermophilus* α-amylase(1QHQ); (D) MalS and *Niallia circulans* Cyclomaltodextrin glucanotransferase(1eo5) alignment. The MalS, *Anoxybacillus ayderensis* α-amylase(5a2a), *Geobacillus stearothermophilus* α-amylase(1QHQ), *Niallia circulans* and Cyclomaltodextrin glucanotransferase(1eo5) are shown in white, green, yellow, and red.

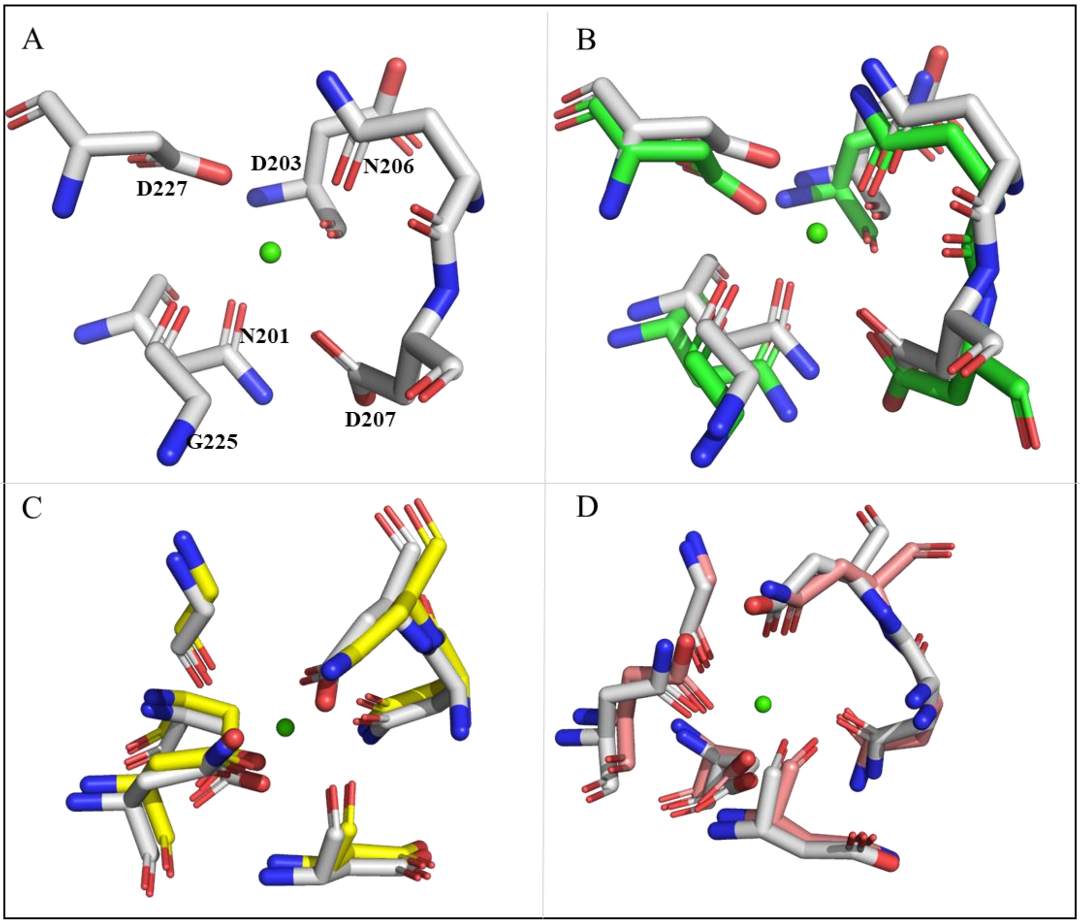

Figure S2B: Ca2 residues alignment. (A) Ca2 interacts with residue of MalS. (B) MalS and *Anoxybacillus ayderensis* α-amylase(5a2a) alignment; (C) MalS and *Geobacillus stearothermophilus* α-amylase(1QHQ); (D) MalS and *Niallia circulans* Cyclomaltodextrin glucanotransferase(1eo5) alignment. The MalS, *Anoxybacillus ayderensis* α-amylase(5a2a), *Geobacillus stearothermophilus* α-amylase(1QHQ), *Niallia circulans* and Cyclomaltodextrin glucanotransferase(1eo5) are shown in white, green, yellow, and red.

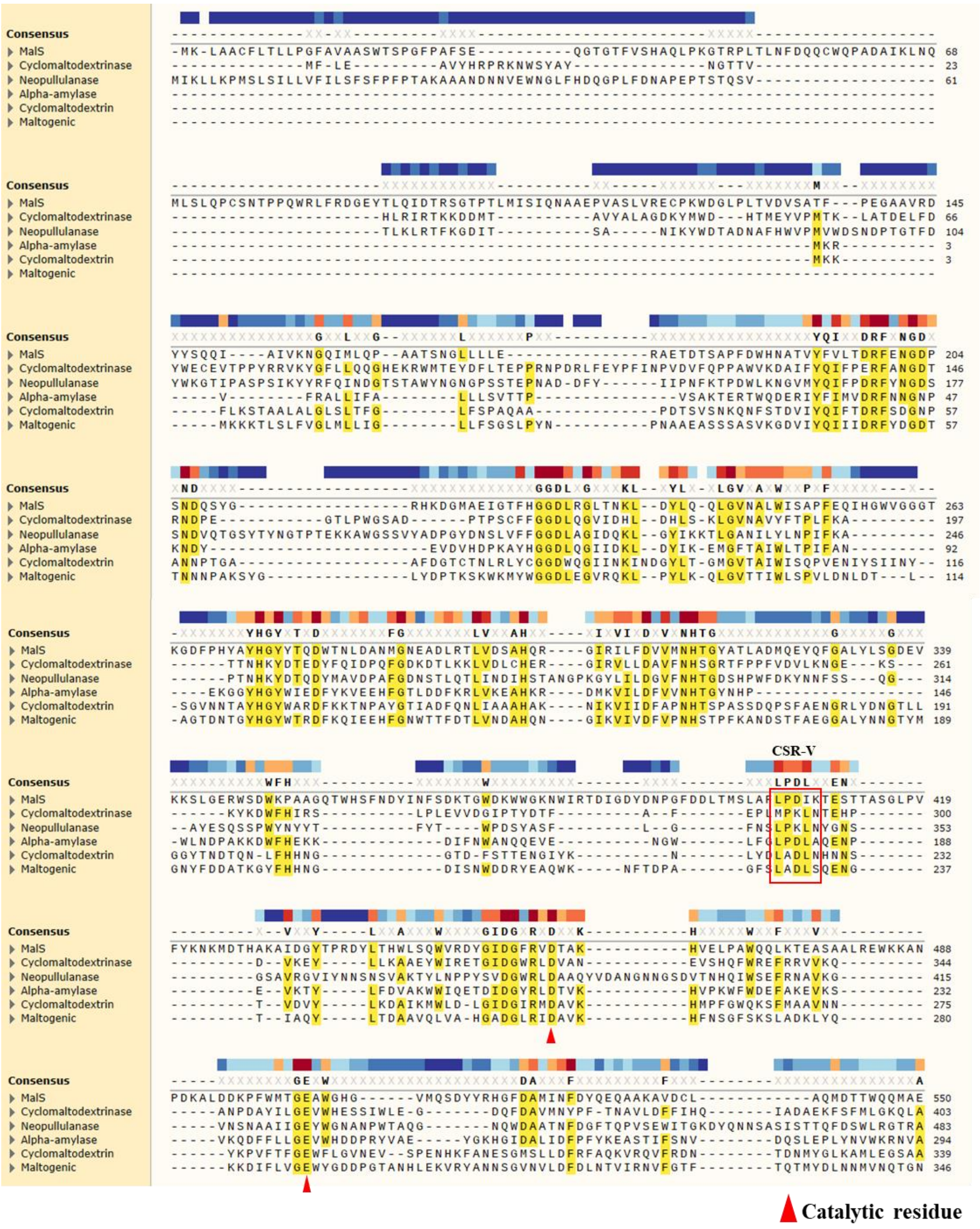

Figure S3A: Multiple sequence alignment of MalS, Cyclomaltodextrinase, Neopullulanase 1, Alpha-amylase (5a2a), Cyclomaltodextrin glucanotransferase, and Maltogenic alpha-amylase.

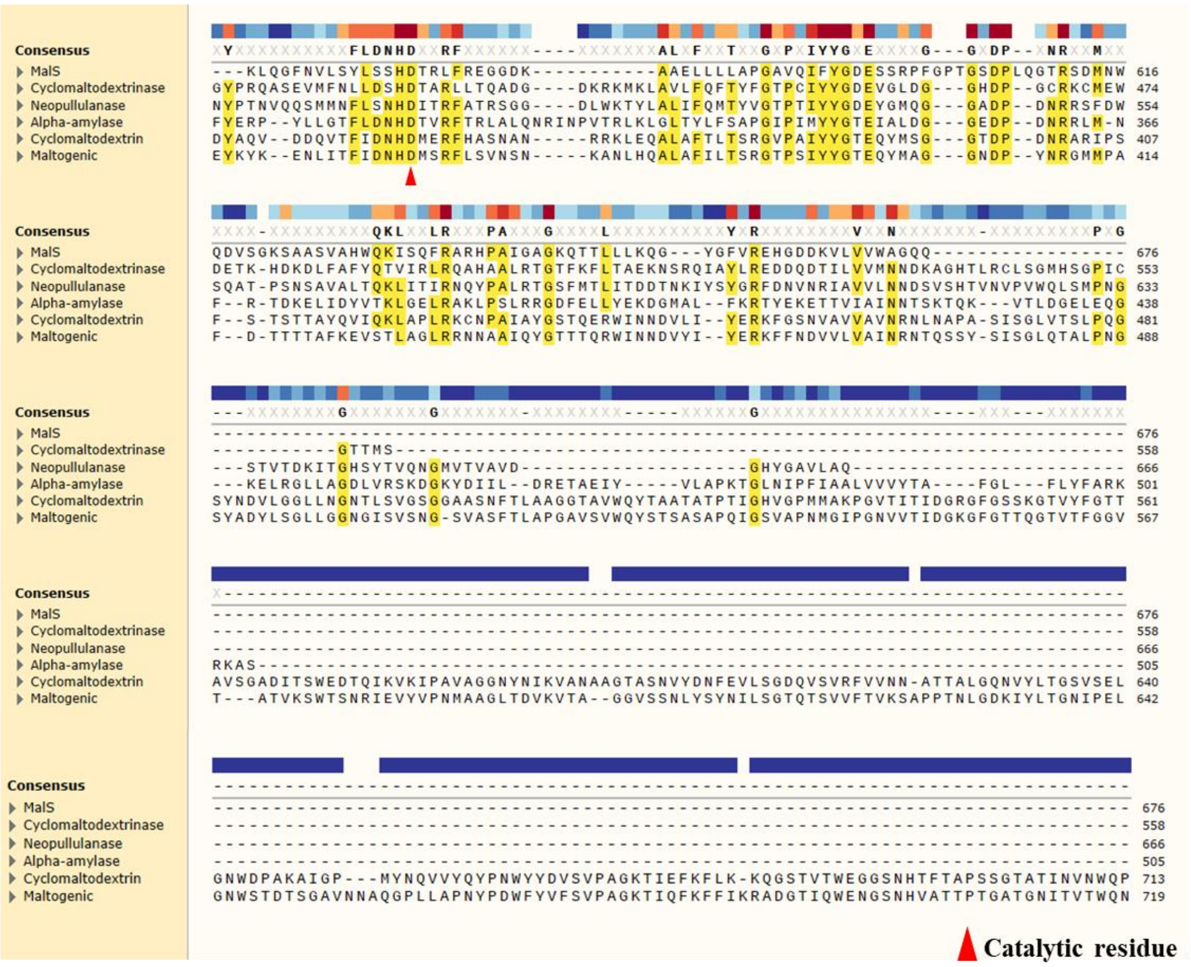

Figure S3B: Multiple sequence alignment of MalS, Cyclomaltodextrinase, Neopullulanase 1, Alpha-amylase (5a2a), Cyclomaltodextrin glucanotransferase, and Maltogenic alpha-amylase.

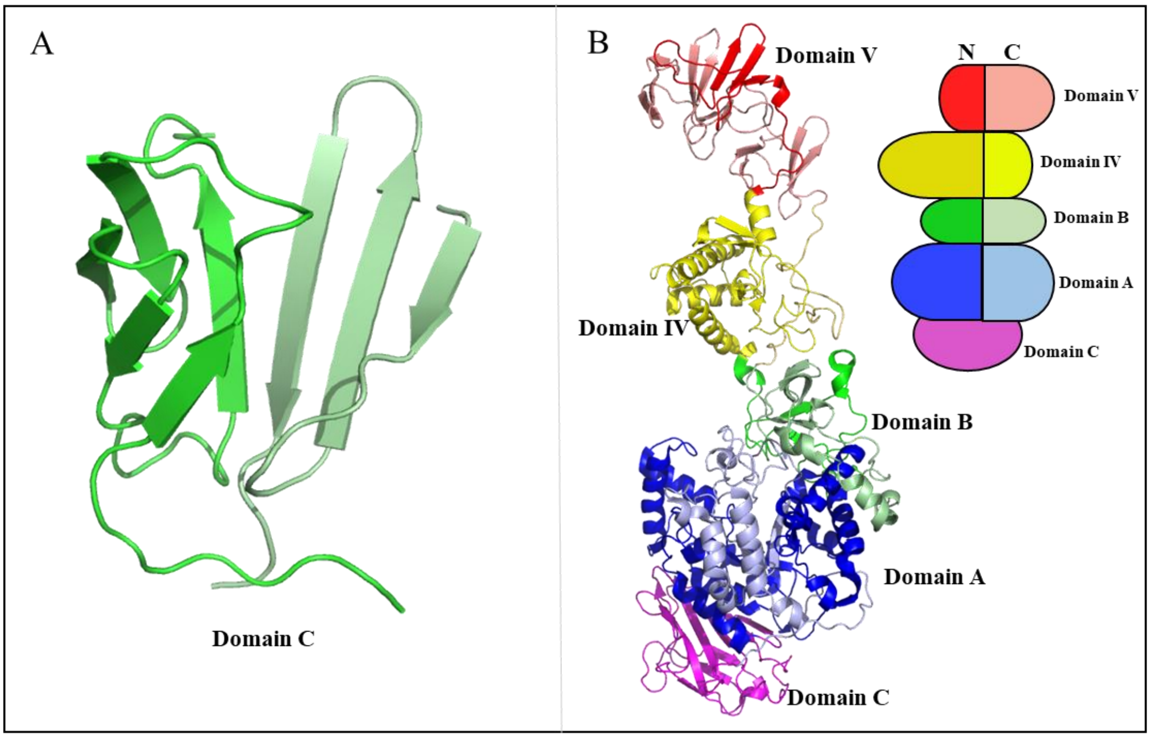

Figure S4: Combined domain. (A) MalS(GH13_19) C domain, with dark and light green colors for the N- and C-terminal stretches of the peptide chain; (B) GTF180-ΔN (glucansucrase, Lactobacillus reuteri 180, PDB: 3KLK, GH70) domains A, B, C, IV, and V are colored in blue, green, magenta, yellow, and red, respectively, with dark and light colors for the N- and C-terminal stretches of the peptide chain. Four(B, C, IV, V) of the five domains are organized in discontinuous parts of the polypeptide chain, similar to MalS C domain, and only the C domain is formed from one continuous stretch of amino acids.

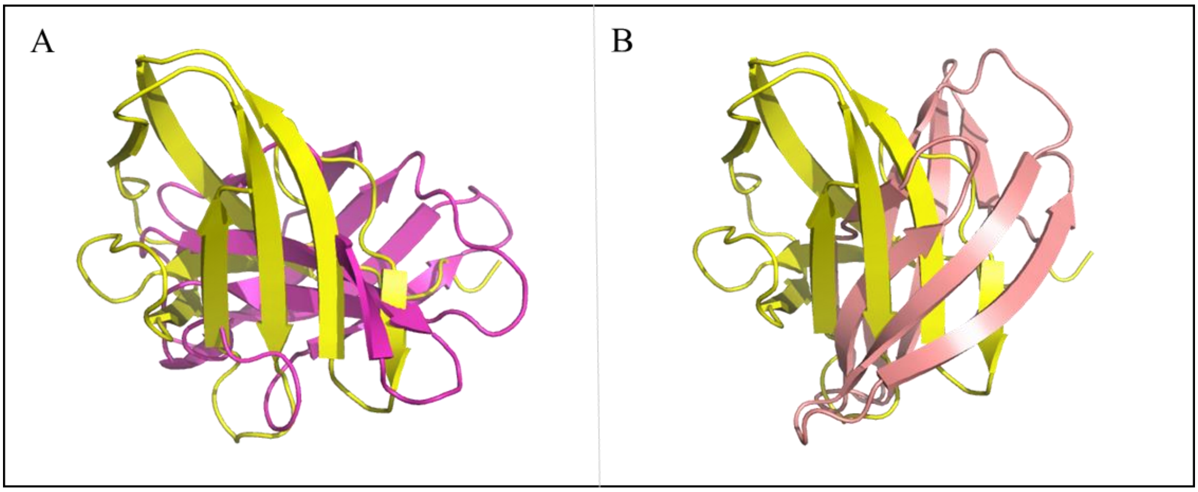

Figure S5: Structure alignment of the Alphafold2-predicted N domain with CBM20 (PDB: 1B90) and CBM48 (PDB: 4AEF). (A) The alignment between the AlphaFold-predicted N domain and CBM20 is shown, with the predicted N domain in yellow and CBM20 in magenta. (B) The alignment between the AlphaFold-predicted N domain and CBM48 is shown, with the predicted N domain in yellow and CBM48 in salmon.

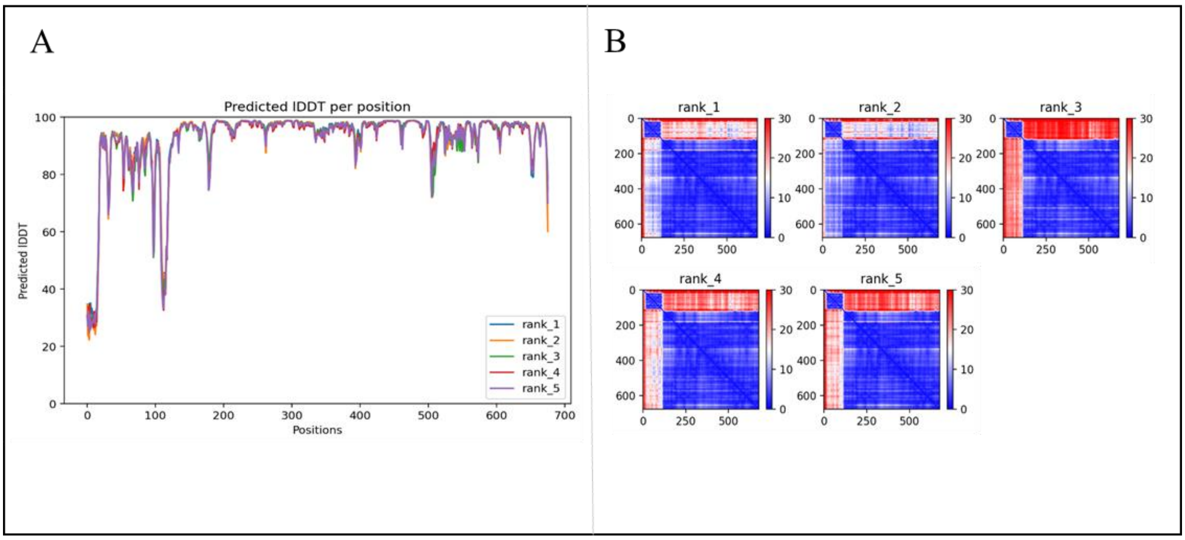

Figure S6: Alphafold2-predicted information of MalS. (A) Predicted per-residue local distance difference test (pLDDT) score for the five models generated by AlphaFold2. The amino acid position is plotted against the predicted pLDDT score. A pLDDT score between 70 and 90 indicates high accuracy, where the prediction of the protein's main chain is reliable. A score above 90 indicates very high accuracy, equivalent to structures determined by experiments. Scores between 50 and 70 indicate lower accuracy, but it is likely that the predictions of individual secondary structures are correct; (B) Prediction aligned error (PAE) score for models. The Prediction Aligned Error (PAE) score displays the calculated error of the predicted distance for each pair of residues. Both axes indicate the position of the individual amino acids. The uncertainty in the predicted distance between two amino acids is shown in the right bar and is color-coded from blue (0 Å) to red (30 Å). The color of the intersection of a horizontal line drawn from the position of an amino acid on the y-axis and a vertical line from the position of another amino acid on the x-axis indicates the error in the predicted distance between these two residues.
